# Diet-mediated constitutive induction of novel IL-4^+^ ILC2 cells maintains intestinal homeostasis in mice

**DOI:** 10.1084/jem.20221773

**Published:** 2023-05-10

**Authors:** Wanlin Cui, Yuji Nagano, Satoru Morita, Takeshi Tanoue, Hidehiro Yamane, Keiko Ishikawa, Toshiro Sato, Masato Kubo, Shohei Hori, Tadatsugu Taniguchi, Masanori Hatakeyama, Koji Atarashi, Kenya Honda

**Affiliations:** 1 Department of Microbiology and Immunology, School of Medicine, Keio University, Tokyo, Japan; 2 RIKEN Center for Integrative Medical Sciences (IMS), Yokohama, Japan; 3 Graduate School of Medicine, The University of Tokyo, Tokyo, Japan; 4 Laboratory of Cellular and Molecular Biology, Center for Cancer Research, National Cancer Institute, National Institutes of Health, Bethesda, MD, USA; 5 Department of Organoid Medicine, Sakaguchi Laboratory, School of Medicine, Keio University, Tokyo, Japan; 6 Institute of Industrial Science, The University of Tokyo, Tokyo, Japan; 7 Division of Molecular Pathology, Research Institute for Biomedical Science, Tokyo University of Science, Noda, Japan; 8 Graduate School of Pharmaceutical Sciences, The University of Tokyo, Tokyo, Japan; 9 Department of Pediatrics, The First Hospital of China Medical University, Shenyang, China; 10 Institute of Microbial Chemistry (BIKAKEN), Microbial Chemistry Research Foundation, Tokyo, Japan.; 11 Center of infection-associated cancer, Institute for Genetic Medicine, Hokkaido University, Sapporo, Japan; 12 Human Biology-Microbiome-Quantum Research Center (WPI-Bio2Q), Keio University, Tokyo, Japan

## Abstract

Group 2 innate lymphoid cells (ILC2s) expressing IL-5 and IL-13 are localized at various mucosal tissues and play critical roles in the induction of type 2 inflammation, response to helminth infection, and tissue repair. Here, we reveal a unique ILC2 subset in the mouse intestine that constitutively expresses IL-4 together with GATA3, ST2, KLRG1, IL-17RB, and IL-5. In this subset, IL-4 expression is regulated by mechanisms similar to but distinct from those observed in T cells and is partly affected by IL-25 signaling. Although the absence of the microbiota had marginal effects, feeding mice with a vitamin B1-deficient diet compromised the number of intestinal IL-4^+^ ILC2s. The decrease in the number of IL-4^+^ ILC2s caused by the vitamin B1 deficiency was accompanied by a reduction in IL-25–producing tuft cells. Our findings reveal that dietary vitamin B1 plays a critical role in maintaining interaction between tuft cells and IL-4^+^ ILC2s, a previously uncharacterized immune cell population that may contribute to maintaining intestinal homeostasis.

## Teaser

A novel ILC2 subset was identified in the mouse intestine that constitutively expresses IL-4. Although the absence of the gut microbiota had marginal effects, feeding mice with a vitamin B1-deficient diet compromised the number of intestinal IL-4^+^ ILC2s.

## Graphical Abstract

**Figure:**
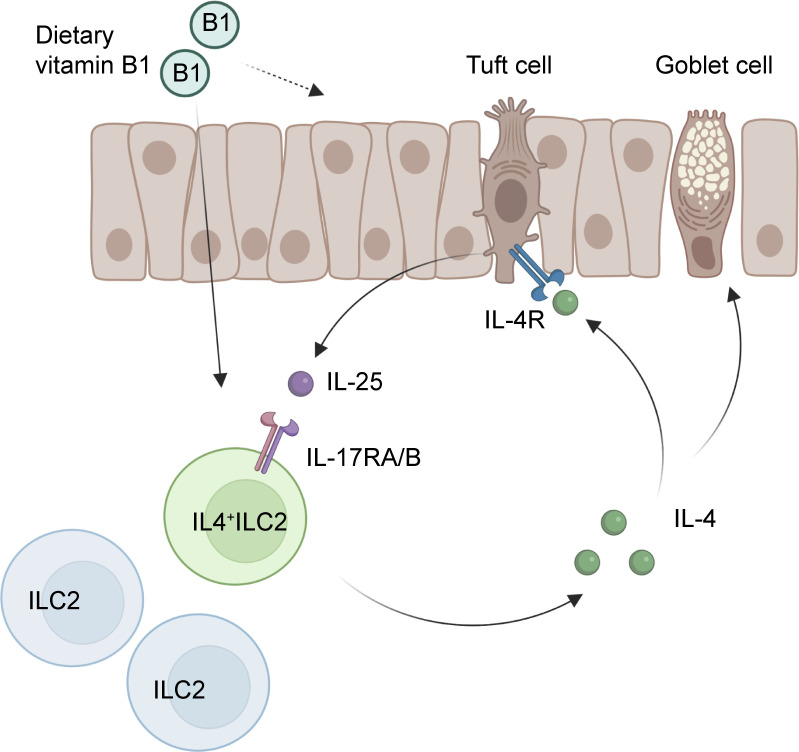

## Introduction

Group 2 innate lymphoid cells (ILC2s) are predominantly localized at mucosal tissues, including skin, lungs, and the gastrointestinal tract, and play critical roles as critical sentinels against infection and tissue damage. In particular, ILC2s serve as the major effector cells against helminth infection (Artis and Spits, 2015; Fallon et al., 2006; Huang et al., 2018; Price et al., 2010). On the other hand, aberrant activation of ILC2s has been implicated in the pathogenesis of allergic diseases, such as asthma (Angkasekwinai et al., 2007; Cavagnero and Doherty, 2017). ILC2s are a subset of Thy1 (CD90)-positive and leukocytes lineage markers (Lin)-negative innate cells and characterized by the expression of the transcription factor GATA3 and share functional characteristics with T helper 2 (T_H_2) cells (Hoyler et al., 2012; Klein Wolterink et al., 2013; Spits and Di Santo, 2011). Epithelial cell-derived cytokines such as interleukin (IL)-25 and IL-33 play critical roles in the development and function of ILC2s. Indeed, in response to IL-25 and IL-33, ILC2s expand and produce large amounts of type 2 cytokines, especially IL-5 and IL-13 (Salimi et al., 2013; Klein Wolterink et al., 2012). ILC2-derived IL-5 acts as a mediator in the activation of eosinophils, whereas IL-13 induces goblet cell metaplasia and mucus secretion (Campbell et al., 2019; Corren, 2011; Nussbaum et al., 2013). Importantly, compared to IL-5 and IL-13, IL-4 expression in ILC2s has been reported to be more tightly regulated and induced under limited conditions (Huang et al., 2015; Miller et al., 2020; Pelly et al., 2016; Roediger et al., 2013).

Recent studies have shown that ILC2s can be subdivided into at least two subtypes: natural ILC2s (nILC2s) and inflammatory ILC2 (iILC2s; Huang et al., 2015; Miller et al., 2020; Ricardo-Gonzalez et al., 2018). nILC2s are tissue-resident cells that express high levels of ST2 (an IL-33 receptor component). In response to IL-33 stimulation, nILC2s moderately proliferate locally at mucosal tissues and play critical roles in immune protection by producing IL-5 and IL-13. In addition, nILC2s express amphiregulin and contribute to tissue repair (Monticelli et al., 2015; Tsou et al., 2022). On the other hand, iILC2s express high levels of KLRG1 (a C-type lectin receptor) and IL-17RB (an IL-25 receptor component), and expand and transiently appear after helminth infection and recombinant IL-25 administration (Huang et al., 2015; Huang et al., 2018; Ricardo-Gonzalez et al., 2020). The development and function of iILC2s are regulated by basic leucine zipper ATF-like transcription factor (BATF; Miller et al., 2020). iILC2s are migratory cells and mobilize to the mucosal sites upon pathogen exposure.

In the intestine, tuft cells are known to be the major sources of IL-25 secretion. Tuft cells express doublecortin-like kinase 1 (DCLK1), and DCLK1^+^ tuft cells constitute a very small fraction of epithelial cells (∼0.5% of intestinal epithelial cells; Gerbe et al., 2011). During acute helminth infection, tuft cells produce IL-25 and promote their own hyperplasia via a positive feedback loop, which is critical for helminth clearance (Gerbe et al., 2016; Howitt et al., 2016; von Moltke et al., 2016). In this feed-forward loop, tuft cell-derived IL-25 is known to induce IL-13 production in iILC2s in the intestinal lamina propria (LP). IL-13 signaling then promotes lineage commitment of undifferentiated epithelial progenitors toward goblet and tuft cells. Goblet cell hyperplasia and increased release of mucus contribute to worm expulsion from the gut (Desai et al., 2021; Gerbe et al., 2016; von Moltke et al., 2016). The development of tuft cells is also controlled by environmental factors, such as dietary succinate. Succinate receptor 1 (Sucnr1) is specifically expressed by tuft cells but not other types of intestinal epithelial cells, and succinate induces tuft and goblet cell hyperplasia via Sucnr1 (Lei et al., 2018; Nadjsombati et al., 2018; Schneider et al., 2018).

Vitamin B1 (VB1, also known as thiamine) is a water-soluble vitamin that serves as a co-factor in the metabolism of carbohydrate and amino acids to produce energy and maintain physiological homeostasis. Because mammals do not possess a biosynthetic pathway for VB1, they must obtain this vitamin from commensal microbes or through dietary supplementation (Rodionov et al., 2019). Without exogenous intake of VB1, serious complications such as neurological abnormalities and congestive heart failure can occur (DiNicolantonio et al., 2018). Further, VB1 has been implicated in controlling the function of immune cells and immunometabolism (Peterson et al., 2020).

In this study, we found that a substantial proportion of ILC2s in the intestine, but not other organs, in mice constitutively expressed IL-4. Much like iILC2s, IL-4-expressing ILC2s (hereafter IL-4^+^ ILC2s) expressed IL-17RB and were at least in part dependent on IL-25 signaling for their development. Moreover, IL-4^+^ ILC2s and IL-25–expressing tuft cells were significantly affected by the deficiency of dietary VB1. Therefore, dietary VB1 is required for the maintenance of intestinal tuft cells and the constitutive induction of IL-4^+^ ILC2s. Our study revealed a critical role of diet–epithelial–immune interactions in inducing a previously undescribed immune cell subset.

## Results

### IL-4^+^ ILC2s are abundant in the intestinal LP

**Figure 1.**
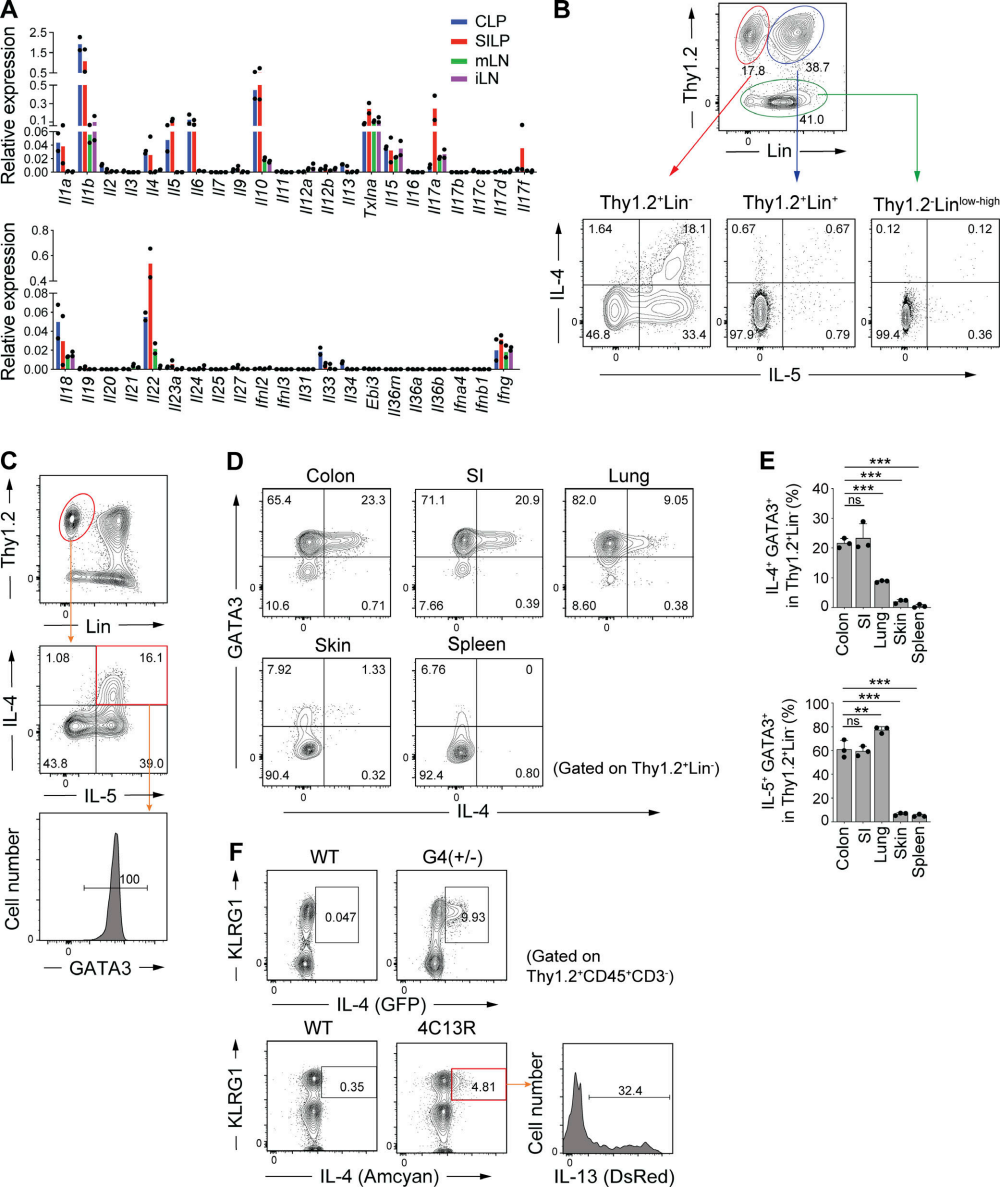
**IL-4**^**+**^ **ILC2s are abundant in the colonic LP. (A)** Expression of the indicated cytokine genes normalized to *Gapdh* in lymphocytes from colonic LP (CLP), small intestinal LP (SILP), mesenteric LN (mLN), and inguinal LN (iLN), as quantified using qPCR. **(B)** Representative flow cytometry plots showing IL-4 and IL-5 expression in three different subsets (Thy1.2^+^Lin^−^, Thy1.2^+^Lin^+^, Thy1.2^−^) of colonic LP lymphocytes stimulated with PMA and ionomycin. **(C)** GATA3 expression in Thy1.2^+^Lin^−^ IL-4^+^ IL-5^+^ cells in colonic LP. **(D)** Representative flow cytometry plots showing the expression of GATA3 and IL-4 by gated Thy1.2^+^Lin^−^ cells in the indicated organs. **(E)** Frequencies of IL-4^+^GATA3^+^ cells and IL-5^+^GATA3^+^ cells among Thy1.2^+^Lin^−^ cells in the indicated organs. Bar graphs showed the mean ± SD. ***P < 0.001; **P < 0.01; ns, not significant; one-way ANOVA with Tukey’s test. Each dot represents an individual mouse. **(F)** Representative flow cytometry plots and histogram showing KLRG1, IL-4, and IL-13 expression by gated colonic LP CD45^+^ Thy1.2^+^CD3^−^ cells. Data shown are representative of more than two independent experiments.

We first assessed cytokine expression profiles in immune cell fractions (Percoll enriched cells) in the small intestinal (SI) and colonic LP and compared them with those in inguinal lymph node (iLN) and mesenteric lymph node (mLN) from specific pathogen-free (SPF; helminth-free) C57BL/6 (B6) mice using RT-qPCR. As expected, T_H_17-, Treg-, and ILC3-related cytokines such as IL-1β, IL-6, IL-10, IL-17A, and IL-22 were found to be expressed at high levels in cells from SI and colonic LP. Notably, substantial levels of IL-4 and IL-5 were also detected specifically in the SI and colonic cells (Fig. 1 A). We thus explored the cellular sources of IL-4 and IL-5 in the intestine. LP cells from SI and colon of SPF mice were stimulated ex vivo with phorbol myristate acetate (PMA) and ionomycin and subjected to intracellular staining for IL-4 and IL-5. Although there were a small number of cells expressing IL-4 and IL-5 among the lineage (Lin)^+^ cells (likely Th2 cells), expression of IL-4 and -5 was mainly observed in Thy1.2^+^Lin^−^ innate cells (Fig. 1 B). IL-4–expressing cells were found to be within the IL-5^+^ cell pool, and IL-4^+^IL-5^+^ double positive cells all exhibited GATA3 expression (Fig. 1 C), indicating that intestinal IL-4^+^IL-5^+^ cells are a subset of ILC2s.

Flow cytometry performed using lymphocytes isolated from various organs of SPF B6 mice revealed that IL-4^+^ ILC2s (GATA3^+^ Thy1.2^+^Lin^−^ cells) were most abundant in the colon and SI, followed by lungs, and were rare in the skin and lymphoid organs, such as spleen (Fig. 1 D). On the contrary, the proportion of IL-5^+^ cells among GATA3^+^ ILC2s was higher in the lung than in the intestine (Fig. 1 E). Examination of two lines of IL-4 reporter mice (4C13R and G4 mice) revealed that a substantial number of intestinal ILC2s constitutively expressed IL-4 without ex vivo stimulation (Fig. 1 F). We also found that a subset of IL-4^+^ cells expressed IL-13 (DsRed; Fig. 1 F). These results indicated that intestinal environment promotes accumulation of IL-4^+^ ILC2s under steady-state conditions.

### Characteristics of colonic IL-4^+^ ILC2s

**Figure 2.**
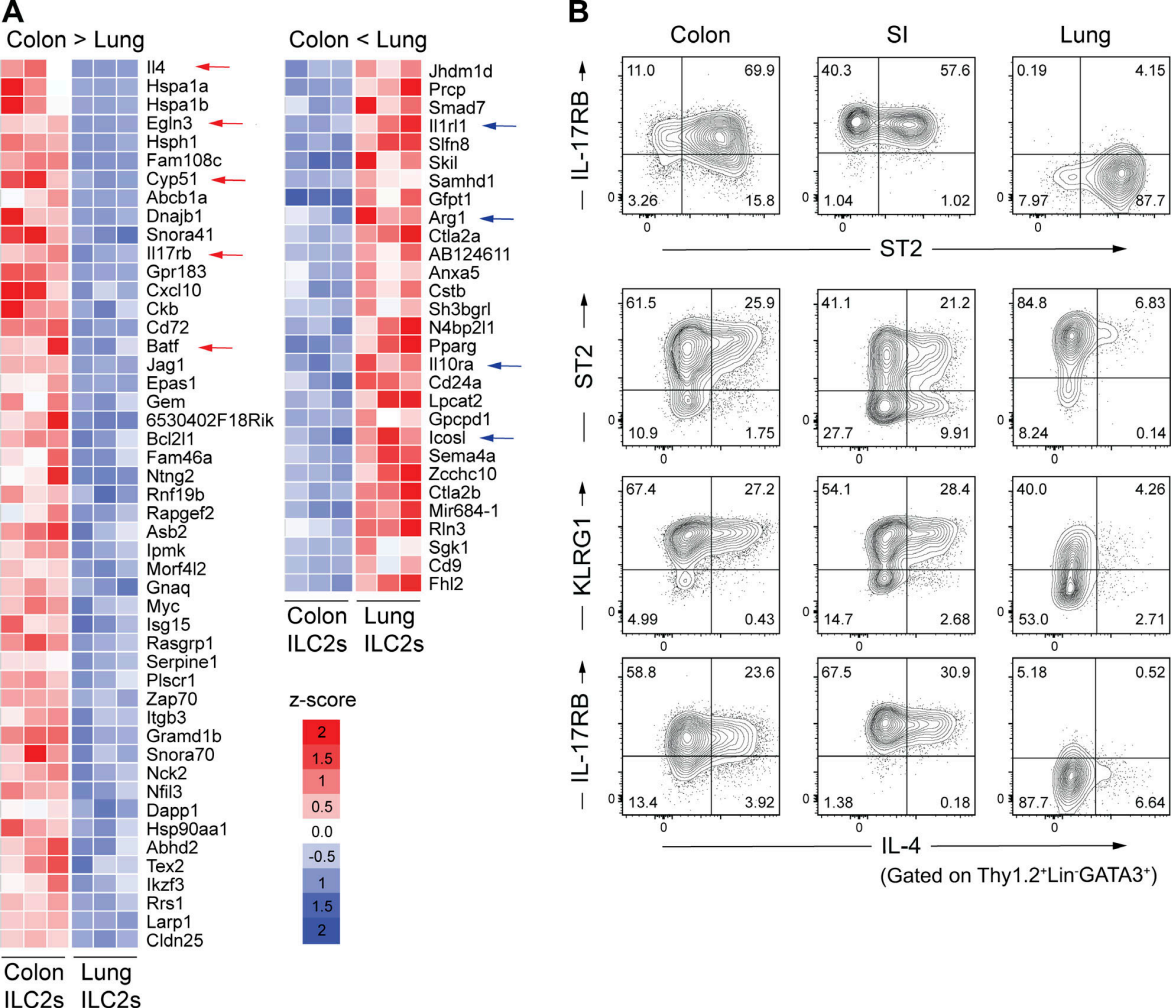
**Characteristics of colonic IL-4**^**+**^ **ILC2s. (A)** Differential gene expression in colonic ILC2s (Thy1.2^+^CD3^−^ KLRG1^+^ cells) and pulmonary ILC2s (Thy1.2^+^CD3^−^ ST2^+^ cells) sorted from SPF B6 mice. Heatmap colors represent the z-score normalized FPKM values for each gene. **(B)** Representative flow cytometry plots showing the expression of IL-17RB, KLRG1, ST2, and IL-4 by gated Thy1.2^+^Lin^−^GATA3^+^ cells in the colon, SI LP, and lungs. Data shown are representative of more than two independent experiments with *n* ≥ 3 individual mice per group.

To further characterize intestinal ILC2s, we compared transcriptomes of colonic LP ILC2s (Thy1^+^ CD3^−^ KLRG1^+^ cells) and pulmonary ILC2s (Thy1^+^ CD3^−^ ST2^+^ cells) through RNA-sequencing (RNAseq). Colonic and pulmonary ILC2s exhibited distinct transcriptional profiles (Fig. 2 A). *Il4* was among the most highly differentially expressed genes in colonic ILC2s compared to that in pulmonary ILC2s (Fig. 2 A). In addition to *Il4*, colonic ILC2s exhibited an enrichment in genes associated with phenotypic markers of iILC2s, such as *Il17rb*, *Klrg1*, and *Batf*, as well as genes related to stress responses, such as those encoding heat shock proteins, a cytochrome P450 superfamily *Cyp51*, and hypoxia-related *Egln3* (Fig. 2 A). In contrast, pulmonary ILC2s expressed high levels of nILC2-associated genes, such as *Il1rl* (St2), *Arginase 1* (*Arg1*), *Il10ra*, and *Icosl* (Fig. 2 A). These findings further suggest that gut and lung environmental factors differentially promote the development of distinct subsets of ILC2s.

To further characterize intestinal IL-4^+^ ILC2s, we conducted additional flow cytometry analyses using anti-ST2, -IL-17RB, and -KLRG1 antibodies. These analyses confirmed differential subpopulations between colonic and lung ILC2s. Colonic GATA3^+^ ILC2s were primarily positive for IL-17RB, ST2, and KLRG1, whereas lung ILC2s were mostly positive for ST2 but negative for IL-17RB and comprised KLRG1 positive and negative subsets that were approximately equal in size (Fig. 2 B). In the colon, the major source of IL-4 was the IL-17RB positive cell subset, whereas IL-4 expression in the lungs was observed within the IL-17RB negative cell subset (Fig. 2 B). We also examined the IL-4^+^ ILC2 population in the SI and found that its characteristics were essentially the same as those of colon IL-4^+^ ILC2s except for the presence of ST2^−^ IL-4^+^ ILC2s (Fig. 2 B).

### IL-25 dependence of colonic IL-4^+^ ILC2s

**Figure 3.**
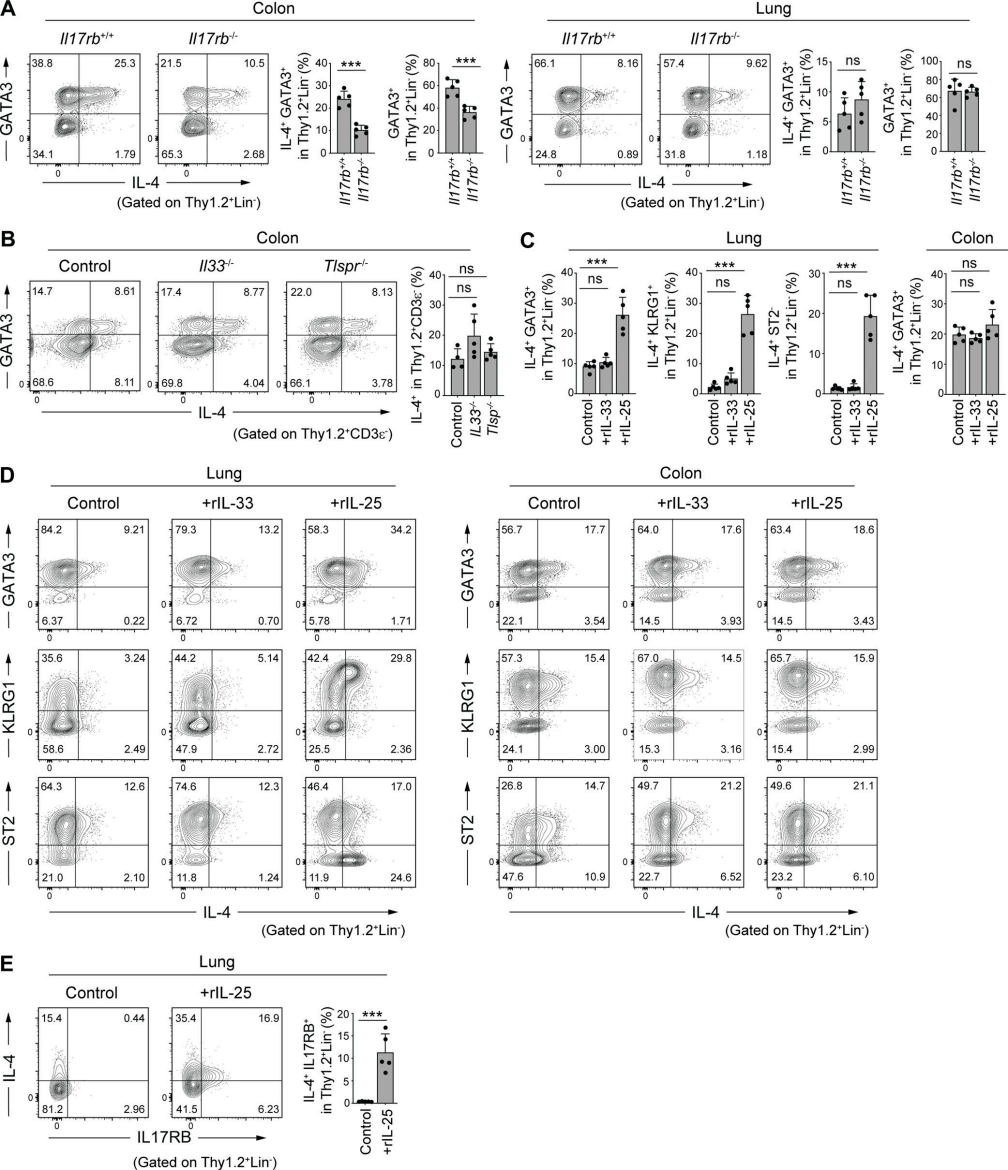
**IL-25 dependence of colonic IL-4**^**+**^ **ILC2s. (A)** Representative flow cytometry plots and frequencies of IL-4^+^GATA3^+^ cells and GATA3^+^ cells among Thy1.2^+^Lin^−^ cells in the colonic LP and lungs of *Il17rb*^+/+^ and *Il17rb*^−/−^ mice. **(B)** Representative flow cytometry plots and frequencies of IL-4^+^ cells among Thy1.2^+^CD3ε^−^ cells in the colonic LP of WT, *Il33*^−/−^, and *Tslpr*^−/−^ mice (BALB/c background). **(C–E)** SPF B6 mice were intraperitoneally injected once daily for 3 d with PBS control, recombinant IL-25 (200 ng/mouse/d), or recombinant IL-33 (200 ng/mouse/d). Frequencies of IL4^+^GATA3^+^, IL-4^+^KLRG1^+^, and IL-4^+^ST2^−^ cells among Thy1.2^+^Lin^−^ cells in the lung and IL4^+^GATA3^+^ cells among Thy1.2^+^Lin^−^ cells in the colon (C), and representative flow cytometry plots showing the expression of IL-4, GATA3, KLRG1, and ST2 by gated Thy1.2^+^Lin^−^ cells from the lungs and colon (D). Representative flow cytometry plots and frequencies of IL-4^+^ IL-17RB^+^ cells in gated Thy1.2^+^Lin^−^ cells from the lungs of mice injected with rIL-25 are shown in E. Bar graphs show the mean ± SD. ***P < 0.001; ns, not significant; two-tailed unpaired Student’s *t* test (A and E), one-way ANOVA with Tukey’s test (B and C). Each dot represents an individual mouse. Data shown are representative of more than two independent experiments with *n* ≥ 4 individual mice per group.

**Figure S1.**
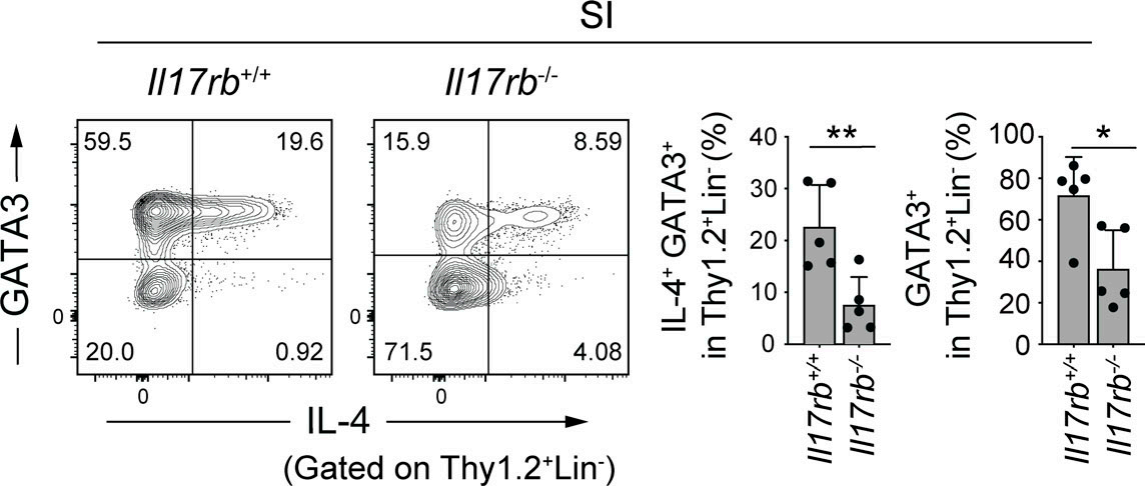
**IL-4**^**+**^ **ILC2s in the small intestine of *Il17rb***^**−/−**^ **mice.** Representative plots (left) and frequencies (right) of IL-4^+^GATA3^+^ cells and GATA3^+^ cells among Thy1.2^+^Lin^−^ cells in the SI LP of *Il17rb*^+/+^ and *Il17rb*^−/−^ mice. Bar graphs show the mean ± SD. **P < 0.01; *P < 0.05; two-tailed unpaired Student’s *t* test. Each dot represents an individual mouse. Data shown are representative of more than three independent experiments with *n* ≥ 3 individual mice per group.

As colonic IL-4^+^ ILC2s were found to express high levels of IL-17RB, we next examined the contribution of IL-25 signaling to the development of intestinal and lung IL-4^+^ ILC2s using *Il17rb*^−/−^ mice. The *Il17rb* deficiency resulted in a partial but significant reduction in the frequency of colonic and SI IL-4^+^ ILC2s, whereas the frequency of lung IL-4^+^ ILC2s was unchanged (Fig. 3 A and Fig. S1). Notably, there was a decrease in the frequency of GATA3^+^ ILC2s in the colon of *Il17rb*^−/−^ mice as compared with wild-type mice, suggesting that IL-25 is involved in the accumulation not specifically of IL-4^+^ ILC2s but more broadly of GATA3^+^ ILC2s in the intestine. As ILC2s are known to be regulated by IL-33 and TSLP, we examined the frequency of colonic IL-4^+^ ILC2s in mice deficient for *Il33* or *Tslp* *receptor* (*Tslpr*). In both *Il33*^−/−^ and *Tslpr*^−/−^ mice (BALB/c background), colonic IL-4^+^ ILC2s were present at similar levels to control BALB/c mice (Fig. 3 B). These results suggest the involvement of IL-25 and undefined factors other than IL-33 and TSLP in the accumulation of intestinal IL-4^+^ ILC2s.

It has been reported that IL-25 administration leads to the induction of an increase in KLRG1^+^ inflammatory ILC2s (iILC2s) in the lungs (Huang et al., 2015; Huang et al., 2018; Ricardo-Gonzalez et al., 2020). Consistent with these reports, intraperitoneal administration of a recombinant IL-25 in SPF B6 mice resulted in a marked increase in the IL-4^+^ KLRG1^+^ GATA3^+^ population of lung innate immune cells (Fig. 3, C and D). In contrast, the administration of recombinant IL-33 did not significantly affect the frequency of IL-4^+^ ILC2s in the lungs (Fig. 3, C and D). The IL-25-induced pulmonary iILC2s exhibited features similar to those seen in colonic IL-4^+^ ILC2s, such as the expression of KLRG1. However, unlike the colonic IL-4^+^ ILC2s that were primarily positive for ST2 (Fig. 2 B), a majority of IL-25-induced lung IL-4^+^ iILC2s were negative for ST2 (Fig. 3 D), consistent with previous reports (Huang et al., 2015). The administration of IL-25 upregulated the expression of IL-17RB in lung ILC2 cells (Fig. 3 E), suggesting that a feed-forward activation of IL-25 and IL-17RB likely leads to the accumulation of IL-4^+^ ILC2s in lungs. Notably, the proportion of colonic IL-4^+^ ILC2s was not significantly affected by the administration of IL-25 (Fig. 3, C and D). Thus, IL-25–induced pulmonary IL-4^+^ ILC2s are likely distinct from colonic constitutive IL-4^+^ ILC2s. It is likely that colonic IL-4^+^ ILC2s are not simply induced by epithelium-derived IL-25 and that additional factors are involved in the induction and maintenance of colonic IL-4^+^ ILC2s.

### *HS2*- and *CNS2*-dependent but BATF-independent induction of colonic IL-4^+^ ILC2s

**Figure 4.**
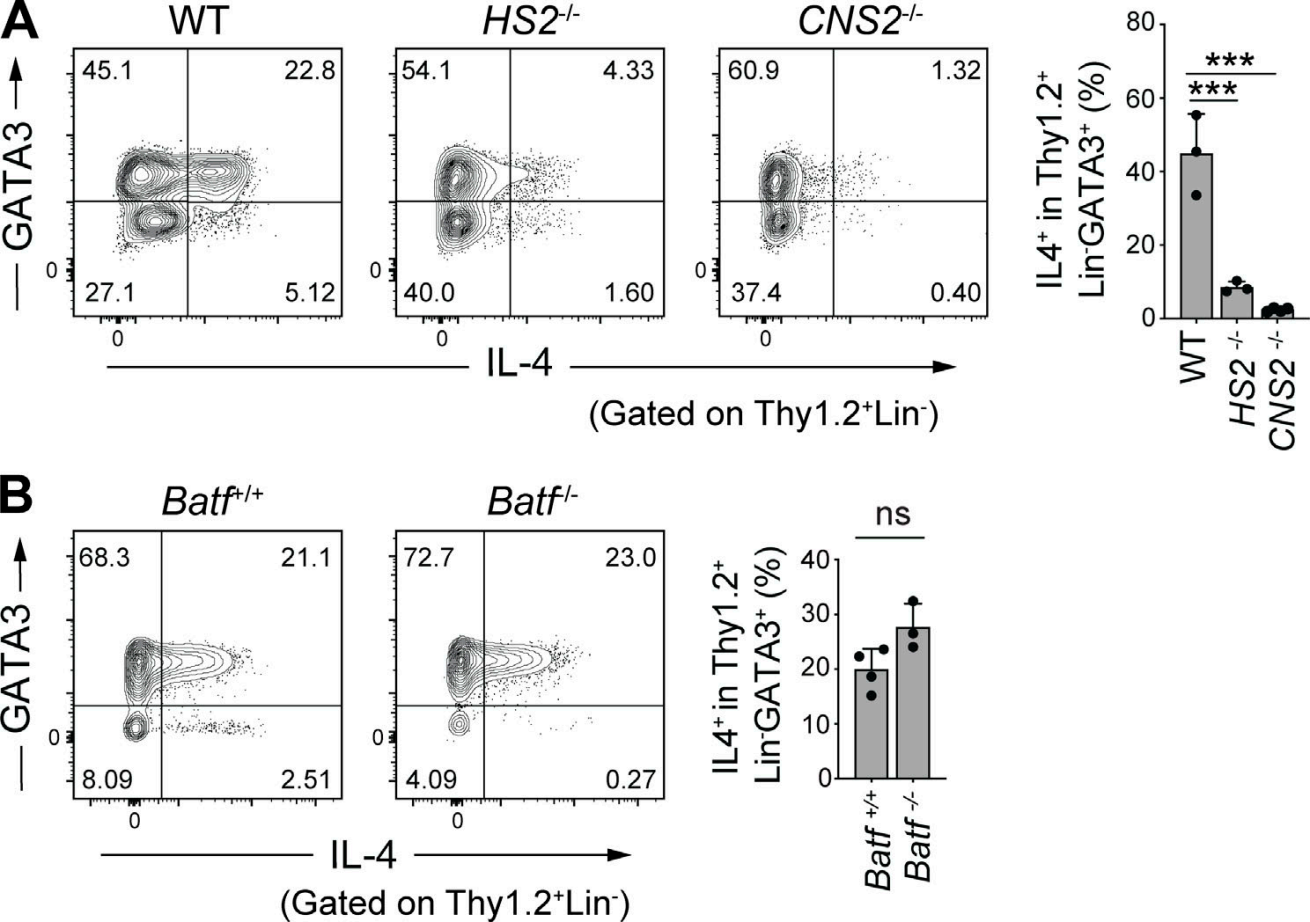
**Unique regulation of *Il4* gene expression in colonic IL-4**^**+**^ **ILC2s. (A)** Representative flow cytometry plots (left) and frequencies (right) of IL-4^+^ cells among Thy1.2^+^Lin^−^GATA3^+^ cells in the colonic LP of mice deficient for the *HS2* or *CNS2* loci of the *Il4* gene. **(B)** Representative flow cytometry plots (left) and frequencies (right) of IL-4^+^ cells among Thy1.2^+^Lin^−^GATA3^+^ cells in the colonic LP of mice deficient for the *Batf* gene. Bar graphs show the mean ± SD. ***P < 0.001; ns, not significant; one-way ANOVA with Tukey’s test (A), two-tailed unpaired Student’s *t* test (B). Each dot represents an individual mouse. Data shown are representative of more than two independent experiments with *n* ≥ 3 individual mice per group.

To address the mechanisms underlying the constitutive expression of IL-4 in intestinal ILC2s, we next examined the involvement of *cis*-regulatory elements in the *Il4* gene locus, using mice deficient for *Il4* intronic enhancer *HS2*, which is a binding site for GATA3 and plays a critical role in IL-4 expression in T_H_2 cells and follicular helper T cells (T_FH_ cells; Tanaka et al., 2011). The *HS2* deficiency resulted in severely reduced IL-4 expression in colonic ILC2s (Fig. 4 A). We also examined mice deficient for the conserved non-coding sequence 2 (*CNS2*) region of *Il4*, which serves as a BATF and RBP-J binding site and plays the role of a T_FH_ cell-specific enhancer (Harada et al., 2012; Vijayanand et al., 2012). We observed a marked reduction in the frequency of colonic IL-4^+^ ILC2s in *CNS2*-deficient mice (Fig. 4 A). Therefore, colonic ILC2s require both the *HS2* and *CNS2* regions to express IL-4, similar to T_FH_ cells.

As BATF plays a critical role in IL-4 expression in T_FH_ cells (Betz et al., 2010; Sahoo et al., 2015), we evaluated the possible involvement of BATF in IL-4 expression in colonic ILC2s. However, *Batf*^−/−^ mice did not exhibit a significant reduction in IL4^+^ ILC2 frequency (Fig. 4 B). These results suggest that IL-4 expression in colonic ILC2s is regulated by a transcriptional program distinct from that of T cells.

### Microbiota-independent, dietary VB1-dependent accumulation of intestinal IL-4^+^ ILC2s

**Figure 5.**
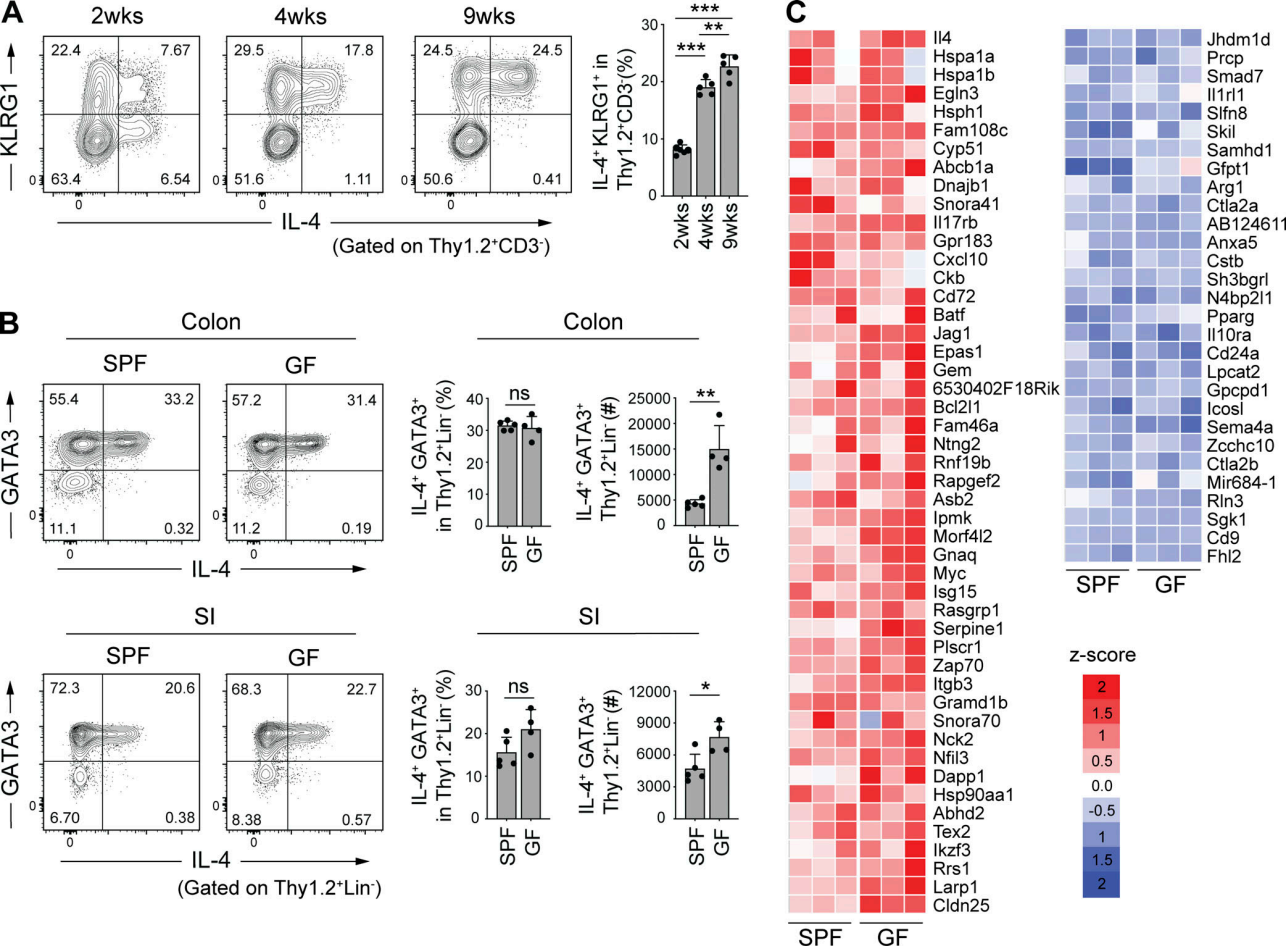
**Microbiota-independent induction of colonic IL-4**^**+**^ **ILC2s. (A)** Representative flow cytometry plots (left) and frequencies (right) of IL-4^+^KLRG1^+^ cells among Thy1.2^+^CD3^−^ cells in the colonic LP of SPF B6 mice at 2–9 wk of age. **(B)** Representative flow cytometry plots (left), frequencies, and absolute number (right) of IL-4^+^GATA3^+^ cells among Thy1.2^+^Lin^−^ cells in the intestinal LP of SPF and GF B6 mice. Bar graphs show the mean ± SD. ***P < 0.001; **P < 0.01; *P < 0.05; ns, not significant; one-way ANOVA with Tukey’s test (A), two-tailed unpaired Student’s *t* test (B). Each dot represents an individual mouse. **(C)** Relative expression of genes listed in Fig. 2 A in colonic ILC2s (Thy1.2^+^CD3^−^KLRG1^+^ cells) sorted from SPF (*n* = 3) and GF (*n* = 3) B6 mice. Heatmap colors represent the z-score normalized FPKM values for each gene. Data shown are representative of more than two independent experiments with *n* ≥ 3 individual mice per group.

We next examined the development of intestinal IL-4^+^ ILC2s during ontogeny. The frequency of IL-4^+^ ILC2s in colonic LP gradually increased until 9 wk of age (Fig. 5 A). In particular, the frequency of the KLRG1^+^ IL-4^+^ ILC2 fraction was greatly increased after 2 wk of age (after weaning; Fig. 5 A). These findings suggest that the intestinal environment provides unique factors for the development and accumulation of IL-4^+^ ILC2s.

We thus explored to elucidate the intestinal factors that may promote the accumulation of IL-4^+^ ILC2s. Given the potentially large impact of the microbiota on the mucosal immune system, we examined the frequency of colonic and SI IL-4^+^ ILC2s in germ-free (GF) mice. The frequencies of colonic and SI IL-4^+^ ILC2s were not significantly different between GF and SPF mice (Fig. 5 B). The absolute number of IL-4^+^ ILC2 was even increased rather than decreased in GF mice (Fig. 5 B). Furthermore, RNAseq analysis of colonic ILC2s (Thy1^+^ CD3^−^ KLRG1^+^ cells) isolated from GF and SPF B6 mice revealed similar gene expression profiles (Fig. 5 C), suggesting that intestinal factors other than gut microbiota are responsible for triggering the development of intestinal IL-4^+^ ILC2s.

**Figure 6.**
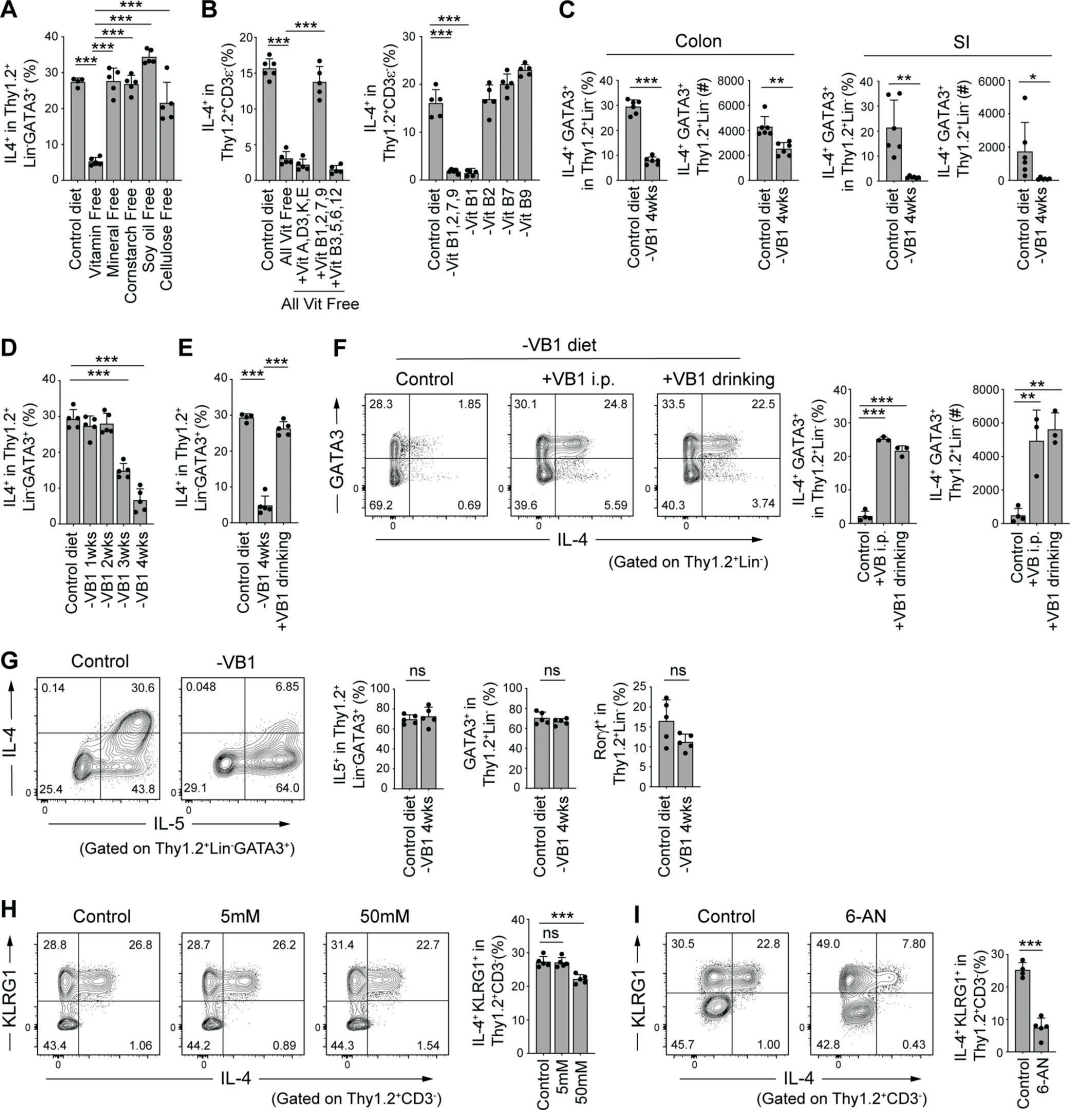
**VB1-dependent induction of colonic IL-4**^**+**^ **ILC2s. (A)** Frequencies of IL-4^+^ cells among Thy1.2^+^Lin^−^GATA3^+^ population in the colonic LP of SPF B6 mice fed a control diet or a diet lacking the indicated dietary components for 4 wk. **(B)** Frequencies of IL-4^+^ cells among Thy1.2^+^ CD3ε^−^ population in the colonic LP of SPF B6 mice fed a control diet, an all vitamin-free diet, a vitamin-free diet supplemented with the indicated vitamins (left), or a diet deprived of the indicated vitamin B component (right). **(C)** Frequencies and absolute number of IL-4^+^GATA3^+^ cells among Thy1.2^+^Lin^−^ cells in colonic and SI LP of SPF B6 mice fed a control diet or VB1-deficient (-VB1) diet. **(D and E)** Frequencies of IL-4^+^ cells among Thy1.2^+^Lin^−^GATA3^+^ population in the colonic LP of SPF B6 mice fed VB1-deficient diet for the indicated weeks (D) or VB1-deficient diet for 4 wk and then supplemented with VB1 for 1 wk via the drinking water (E). **(F)** Representative flow cytometry plots and frequencies of IL-4^+^GATA3^+^ cells among Thy1.2^+^Lin^−^ cells in the colonic LP of SPF B6 mice fed a VB1-deficient diet for 4 wk followed by treatment with VB1 either through i.p. injection or drinking water for 1 wk. **(G)** SPF mice were fed a VB1-deficient diet for 4 wk. Representative flow cytometry plots (left) showing the expression of IL-4 and IL-5 by gated Thy1.2^+^Lin^−^GATA3^+^ cells in the colonic LP and frequencies (right) of colonic LP IL-5^+^ cells among Thy1.2^+^Lin^−^GATA3^+^ population and GATA3^+^ or Rorγt^+^ cells among Thy1.2^+^Lin^−^ population. **(H)** Representative plots (left) and frequencies (right) of IL-4^+^KLRG1^+^ cells among Thy1.2^+^CD3^−^ cells in the colonic LP of SPF B6 mice treated with 0, 5, or 50 mM lactic acid for 5 wk in drinking water. **(I)** Representative plots (left) and frequencies (right) of IL-4^+^KLRG1^+^ cells among Thy1.2^+^CD3^−^ cells in the colonic LP of SPF B6 mice treated with PBS or 6-AN. Bar graphs show the mean ± SD. ***P < 0.001; **P < 0.01; *P < 0.05; ns, not significant; one-way ANOVA with Tukey’s test (A, B, D–F, and H), two-tailed unpaired Student’s *t* test (C, G, and I). Each dot represents an individual mouse. Data shown are representative of more than two independent experiments with *n* ≥ 3 individual mice per group.

In view of the gut microbiota-independent development of colonic IL-4^+^ ILC2s, we next explored the influence of dietary components. SPF mice (8-wk-old) were fed a defined diet devoid of either cellulose, soy oil, corn starch, minerals, or vitamins for 4 wk. Feeding with a diet deficient in vitamins led to a significant reduction in colonic IL-4^+^ ILC2s, whereas deprivation of other dietary components showed marginal effects (Fig. 6 A). We then assessed the involvement of each of the vitamins and found that supplementation of a mixture of vitamin B1, B2, B7, and B9 to the diet deficient in all vitamins ameliorated reduced frequency of colonic IL-4^+^ ILC2s (Fig. 6 B, left). Of vitamins B1, B2, B7, and B9, only a diet deficient in VB1 resulted in a reduction of colonic IL-4^+^ ILC2s (Fig. 6 B, right). The frequency and number of IL-4^+^ ILC2 in the SI were affected following a VB1-depleted diet, similarly to the colon (Fig. 6 C). After feeding a VB1-deficient diet, the decrease in IL-4^+^ ILC2s started at around 3 wk and went on decreasing until 4 wk (Fig. 6 D). The decrease was reversed by supplementation with VB1 in the drinking water for 1 wk (Fig. 6 E). Intraperitoneal injection with VB1 also sufficiently restored the number and frequency of IL-4^+^ ILC2s in the colon (Fig. 6 F), indicating that VB1’s effects are not necessarily dependent on intestinal absorption. As compared with IL-4^+^ ILC2s, the frequencies of GATA3^+^ ILC2s and RORγt^+^ ILC3s among Thy1.2^+^Lin^−^ cells and IL5^+^ cells among GATA3^+^ ILC2s were less affected by VB1-deficiency (Fig. 6 G). These results suggest that VB1 in diets promotes the development and maintenance of intestinal IL-4^+^ ILC2s in an inducible and reversible manner.

VB1 plays a crucial role in carbohydrate metabolism. VB1-deficiency is known to affect the conversion of pyruvate to acetyl-CoA, which is the entry point into the citric acid cycle, resulting in lactate accumulation. Therefore, we investigated whether lactic acid accumulation might be a mechanism by which VB1-deficiency reduced IL-4^+^ ILC2 levels. However, treatment with lactic acid through the drinking water (even at a high concentration) had little impact on colonic IL-4^+^ ILC2 levels (Fig. 6 H). Since VB1 is a co-factor of glucose-6-phosphate dehydrogenase (G6PD) in the pentose phosphate pathway (PPP), we next examined the effect of 6-aminonicotinamide (6-AN), a competitive inhibitor of G6PD, on colonic IL-4^+^ ILC2s (Pelicano et al., 2006; Zastre et al., 2013). Intraperitoneal injections with 6-AN significantly reduced colonic L-4^+^ ILC2s (Fig. 6 I). These findings suggest that VB1 contributes to the maintenance of IL-4^+^ ILC2 possibly by fueling metabolic pathways, including the PPP, in ILC2s.

### Dietary VB1 maintains IL-4^+^ ILC2s and IL-25–producing tuft cells

**Figure 7.**
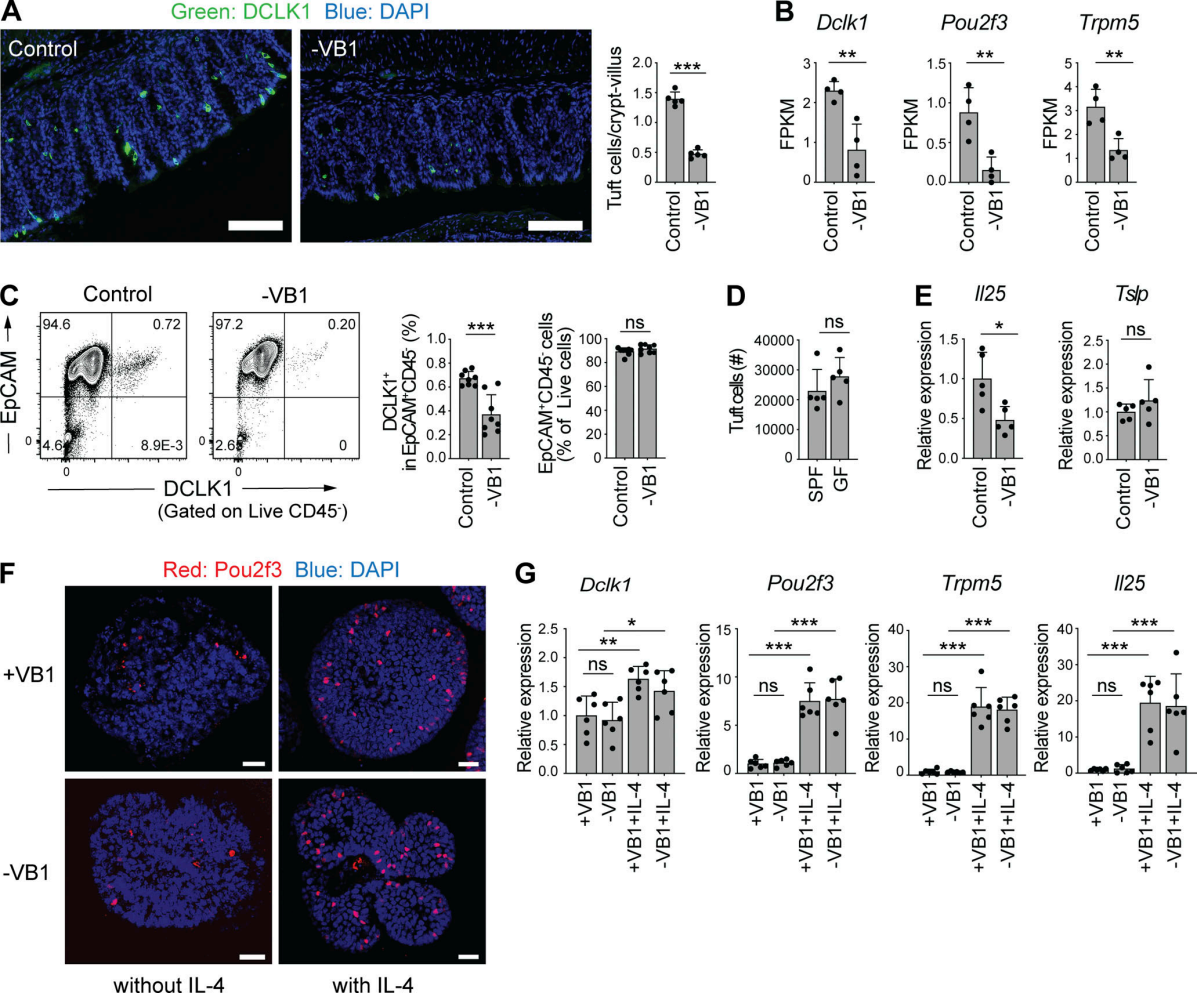
**Dietary VB1 maintains IL-4**^**+**^**ILC2– and IL-25–producing tuft cell populations. (A)** Colon sections from SPF B6 mice fed a control or VB1-deficient diet were stained for DCLK1 (green) and DAPI (blue). Scale bar = 100 μm. Bar graph shows the number of DCLK1^+^ cells per crypt-villus of the colon. **(B)** Expression of the indicated genes in colonic epithelium isolated from SPF C57/B6 mice fed a control or VB1-deficient (-VB1) diet, as quantified by RNAseq. **(C)** Representative flow cytometry plots (left) of colonic tuft cells (EpCAM^+^CD45^−^DCLK1^+^). Bar graphs show frequency of colonic DCLK^+^ cells among CD45^−^EpCAM^+^ population and EpCAM^+^CD45^−^ cells among live cells. **(D)** The number of tuft cells (EpCAM^+^CD45^−^DCLK1^+^) in the colon of GF and SPF B6 mice was measured by flow cytometry. **(E)** qPCR quantification of IL-25 and TSLP expression normalized to *Actb* in the colonic epithelial cell fraction from mice fed a control or VB1-deficient diet. **(F and G)** Colonic organoids from mice were grown in the presence or absence of VB1, followed by incubation with or without recombinant IL-4. Whole-mount immunofluorescent staining of the organoids with anti-Pou2f3 antibody (red) and DAPI (blue) are shown in F. qPCR quantification of the indicated gene expression normalized to *Gapdh* is shown in G. Scale bar = 25 μm. Bar graph shows the mean ± SD. ***P < 0.001; **P < 0.01; *P < 0.05; ns, not significant; two-tailed unpaired Student’s *t* test (A–E), one-way ANOVA with Tukey’s test (G). Each dot represents an individual mouse. Data shown are representative of more than two independent experiments with *n* ≥ 4 individual mice per group.

Since intestinal tuft cells have been reported to play a critical role in ILC2 development (Desai et al., 2021; Howitt et al., 2016; Lei et al., 2018; Nadjsombati et al., 2018; Schneider et al., 2018; von Moltke et al., 2016), we next examined the effect of a VB1-deficient diet on intestinal tuft cells. Immunohistochemistry revealed that the colonic crypts in mice that were on a VB1-deficient diet harbored fewer DCLK1^+^ tuft cells than those in mice on a control diet (Fig. 7 A). Congruently, mRNA expressions of hallmark genes of tuft cells, such as *Dclk1*, POU class 2 homeobox 3 (*Pou2f3*), and transient receptor potential melastatin 5 (*Trpm5*), were significantly decreased in epithelial cells obtained from mice on a VB1-deficient diet (Fig. 7 B). Flow cytometry also revealed a significant reduction in the frequency of DCLK1^+^ tuft cells in the colon, although the total EpCAM^+^CD45^−^ colonocyte number was not significantly changed due to VB1 deficiency (Fig. 7 C). In contrast to VB1-deficiency, the number of tuft cells was not significantly affected by the absence of gut microbiota (Fig. 7 D). As tuft cells have been reported to be one of the major sources of IL-25 (Lei et al., 2018; Nadjsombati et al., 2018; Schneider et al., 2018; von Moltke et al., 2016), we examined IL-25 mRNA levels in the colonic epithelial cells using qPCR. Compared to cells obtained from mice on a control diet, we detected lower IL-25 levels but comparable TSLP levels in the epithelial cells obtained from mice fed a VB1-deficient diet (Fig. 7 E). These results indicate that VB1 is required for the maintenance of tuft cells and local IL-25 levels in the intestine.

**Figure S2.**
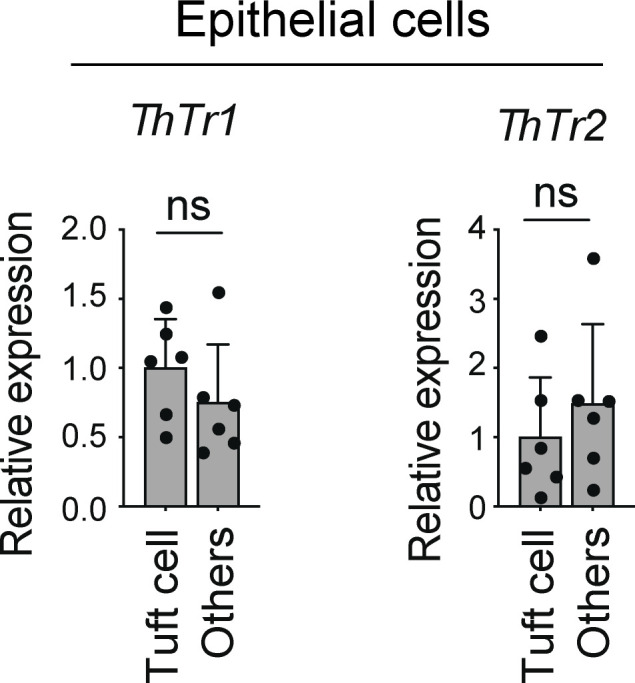
**Expression of VB1 transporters by intestinal epithelial cells.** qPCR quantification of *ThTr1* and *ThTr2* expression normalized to *Actb* in the colonic tuft cells (EpCAM^+^CD45^−^DCLK1^+^) and other (EpCAM^+^CD45^−^DCLK1^−^) epithelial cells isolated from SPF B6 mice by flow cytometry. ns, not significant; two-tailed unpaired Student’s *t* test. Each dot represents an individual mouse. Data shown are representative of more than two independent experiments with ≥3 individual mice per group.

Next, we examined whether VB1 directly affects tuft cell development. To this end, we used an ex vivo organoid culture system derived from mouse colon pluripotent stem cells. The organoids were cultured in the presence or absence of VB1 and subjected to qPCR analysis and immune staining for tuft cell markers. A lack of VB1 did not affect the development of Pou2f3^+^ tuft cells (Fig. 7 F) and the expression of tuft cell signature genes (*Dclk1*, *Pou2f3*, and *Trpm5*). Further, *Il25* was not significantly different between organoids cultured with and without VB1 (Fig. 7 G). We also examined the expression of thiamin transporter-1 (*ThTr1*) and thiamin transporter-2 (*ThTr2*) in DCLK1^+^ and DCLK1^−^ epithelial cells isolated from SPF B6 mice and found that there was no significant difference in their expression between DCLK1^+^ tuft cells and other epithelial cells (Fig. S2). These results suggest that VB1 might influence tuft cells via indirect mechanisms. We, therefore, examined the effect of IL-4 (potentially provided by IL-4^+^ ILC2s) on tuft cell development. To this end, organoids were cultured in the presence of IL-4. The development of Pou2f3^+^ cells was substantially enhanced, and the expression of the tuft cell signature genes and *Il25* was significantly increased in organoids following IL-4 treatment regardless of the presence of VB1 (Fig. 7, F and G). These results suggest that VB1 (potentially together with IL-25) may act primarily on ILC2s, rather than tuft cells, to induce the accumulation of IL-4^+^ ILC2s, and IL-4, in turn, promotes the differentiation of IL-25–expressing tuft cells and increases local levels of IL-25. This feed-forward loop involving VB1, IL-4, and IL-25 created by homeostatic interaction between diet, ILC2s, and tuft cells likely promotes further accumulation of IL-4^+^ ILC2s.

### Exacerbation of experimental colitis by VB1 deficiency

**Figure 8.**
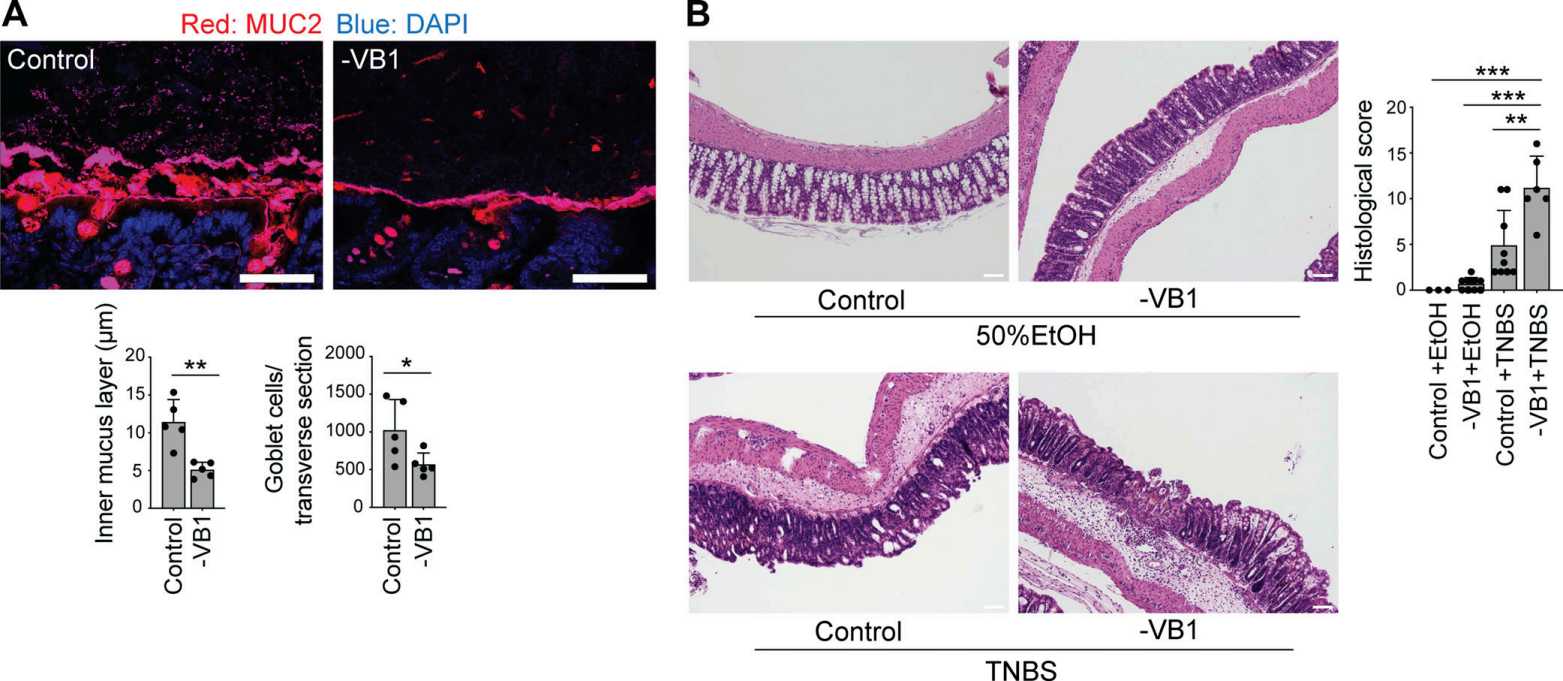
**Exacerbation of experimental colitis by VB1 deficiency. (A)** The proximal colon from SPF B6 mice fed a control or VB1-deficient (-VB1) diet were subjected to fluorescence staining with Muc2 (red) and DAPI (blue; upper panels), and the thickness of the inner mucus layer and the number of goblet cells in transverse sections of each mouse were determined using ImageJ software (lower bar graphs). Scale bar = 50 μm. **(B)** TNBS-colitis was induced in SPF B6 mice fed a control or VB1-deficient diet for 3 wk. H&E staining (left) and histological score (right) of the indicated mice are shown. Scale bar = 100 μm. Scale bar = 100 μm. Bar graph shows the mean ± SD. ***P < 0.001; **P < 0.01; *P < 0.05; two-tailed unpaired Student’s *t* test (A) and one-way ANOVA with Tukey’s test (B). Each dot represents an individual mouse. Data shown are representative of more than two independent experiments with *n* ≥ 3 individual mice per group.

**Figure S3.**
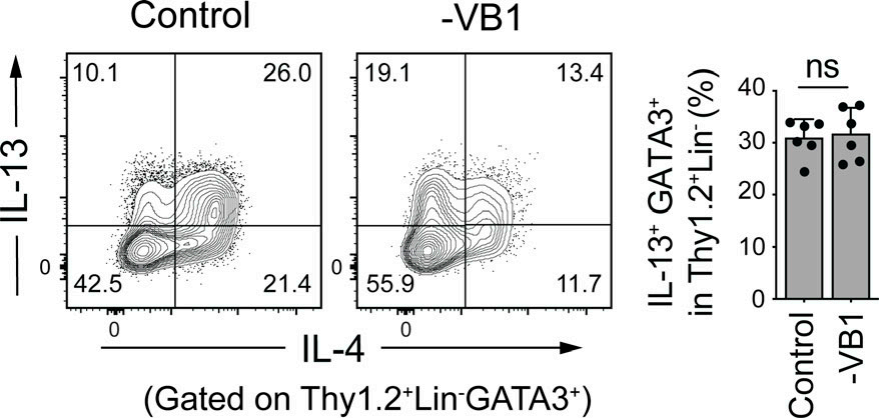
**Influences of VB1-deficiency on IL-13 expression by ILC2s.** Representative plots (left) showing the expression of IL-4 and IL-13 by gated Thy1.2^+^Lin^−^ GATA3^+^ cells and frequencies (right) of IL-13^+^GATA3^+^ cells among Thy1.2^+^Lin^−^ cells in the colonic LP of SPF B6 mice fed a control or VB1-deficient diet. Bar graphs show the mean ± SD. ns, not significant; two-tailed unpaired Student’s *t* test. Each dot represents an individual mouse. Data shown are representative of more than three independent experiments with *n* ≥ 3 individual mice per group.

Finally, we investigated the physiological implications of VB1-mediated induction of tuft cells and IL-4^+^ ILC2s. As ILC2s are critical for controlling goblet cell function, we also evaluated mucin-producing goblet cells and the mucus layer by immunostaining for Muc2. VB1 deficiency led to a significant reduction in mucin-producing goblet cells, accompanied by a thinner mucus layer than that in control mice (Fig. 8 A). Notably, the frequency of colonic ILC2s expressing IL-13, a well-known cytokine that promotes mucin production, was less affected by a VB1-deficient diet (Fig. S3), suggesting the involvement of IL-4 in mucus production. We also subjected VB1-deficient diet-fed mice to trinitrobenzene sulphonic acid (TNBS)-induced colitis, in which IL-4 is reported to contribute to the amelioration of disease (Xiong et al., 2013). The VB1-deficient diet-fed mice were more sensitive to TNBS administration and showed more severe histological disease features than control mice (Fig. 8 B). These results suggest that dietary VB1 plays a critical role in maintaining tuft cells, IL-4^+^ ILC2s, and thus intestinal mucus barrier integrity.

## Discussion

ILC2 cells have been extensively studied in the context of host defense against helminth infection (Pelly et al., 2016; Turner et al., 2013); however, the development and function of ILC2s constitutively residing in the intestine remain unexplored. In the present study, we demonstrated that a unique environment created in the intestines constitutively promotes the development of a new class of ILC2s, which we term IL-4^+^ ILC2 cells, under steady-state conditions. Intestinal IL-4^+^ ILC2s co-express IL-5 and a part of them additionally express IL-13. In organs outside of the intestine, such as the lungs, the majority of ILC2s are nILC2-like cells, expressing ST2, Arg1, and IL-5, with some of these cells expressing low levels of IL-4. In contrast, colonic IL-4^+^ ILC2s express BATF, KLRG1, and IL-17RB and are phenotypically similar to the previously reported IL-25–induced iILC2 cell subset (Huang et al., 2015; Huang et al., 2018; Saenz et al., 2013). However, unlike the IL-25–induced iILC2s in the lungs (the majority of which are negative for IL-17RB and IL-33 receptor component ST2), colonic IL-4^+^ ILC2s constitutively express ST2 at high levels. Additionally, recombinant IL-25 administration failed to increase the number of colonic IL-4^+^ ILC2s, and *Il17rb* deficiency led to a partial reduction in IL-4^+^ ILC2s. The absence of IL-33 or TSLP signaling did not affect the development of intestinal IL-4^+^ ILC2s. Therefore, it is likely that, in addition to IL-25, yet undefined additional factors contribute to the constitutive accumulation of IL-4^+^ ILC2s in the colon.

The development and function of colonic innate and adaptive immune cell populations are strongly influenced by the presence of the microbiota (Gury-BenAri et al., 2016; Honda and Littman, 2016; Tuganbaev and Honda, 2021). However, the frequency of colonic IL-4^+^ ILC2s and their transcriptional profiles were not affected by the absence of the microbiota. We thus honed in on dietary components and found that VB1 was one of the critical intestinal factors affecting the development of colonic IL-4^+^ ILC2s. VB1 deficiency impairs the generation and maintenance of IL-4^+^ ILC2s as well as tuft cells in the colon. It is noteworthy that treatment with a G6PD inhibitor, 6-AN, phenocopied VB1 deficiency. As VB1 works as a co-factor for G6PD, VB1 might affect metabolic pathways, including the PPP, in ILC2s to facilitate the development of IL-4^+^ ILC2s.

Previous studies have shown that diet-derived succinate induces an increase in SI tuft cells (Lei et al., 2018; Schneider et al., 2018). Intestinal luminal succinate engages Sucnr1 on tuft cells and triggers their expansion and subsequent IL-25 production. VB1 acts as a key co-factor for α-ketoglutarate dehydrogenase, which is involved in the conversion of α-ketoglutarate to succinate. Therefore, the action of VB1 might also be linked to succinate-mediated signaling in tuft cells. Our organoid experiment revealed that VB1 was not essential for the development of tuft cells or IL-25 expression in tuft cells. In contrast, IL-4 supplementation significantly enhanced tuft cell differentiation. Consistent with these findings, it has been reported that tuft cells express IL-4R (Gerbe et al., 2016; Haber et al., 2017; Inaba et al., 2021). Therefore, VB1 may promote the development or maintenance of IL-4^+^ ILC2s, which then contribute to the establishment of a feed-forward interaction between ILC2s and tuft cells mediated by IL-4 and IL-25, thereby promoting the generation of both cells. Further investigation is required to understand the mechanism underlying the influence of VB1 on IL-4^+^ ILC2s and tuft cells.

Given the constitutive presence of IL-4^+^ ILC2s in helminth-free healthy mice, they presumably contribute to the maintenance of homeostatic intestinal immune function and intestinal barrier integrity. As VB1-deficiency led to a significant reduction of the thickness of the colonic mucous layer, IL-4^+^ ILC2s may contribute to the induction of goblet cells. Moreover, mice fed a VB1-deficient diet were vulnerable to TNBS-induced colitis. These results suggest that dietary VB1 is essential for maintaining IL-4^+^ ILC2, and, thereby, intestinal mucin production and barrier integrity under both homeostatic and inflammatory conditions. A previous study showed that VB1 affects the development of B cells and IgA antibody responses (Kunisawa et al., 2015). Therefore, VB1 supplementation might help to restore intestinal barrier integrity via inducing B cells and ILC2s, which might be beneficial in treating diseases such as inflammatory bowel disease and parasite infection.

There are a number of limitations to our study. In particular, we could not test the possibility of the direct effect of VB1 on ILC2s, as ILC2s cannot yet be robustly maintained in vitro due to the lack of reliable culture systems. Future investigation and new experimental systems are required to understand the primary target cells and pathways of VB1. Moreover, we were unable to examine the specific functions of IL-4^+^ ILC2s. New tools, such as mice with an ILC2-specific *Il4* gene deficiency or mice specifically lacking the IL-4^+^ ILC2 population, need to be developed to examine the specific role of IL-4^+^ ILC2s. Since it has been reported that ILC2s selectively express the neuromedin U receptor 1 (Nmur1; Klose et al., 2017; Wallrapp et al., 2017) and ILC2-specific Cre strains (Nmur1-Cre mice) have recently been developed (Jarick et al., 2022; Tsou et al., 2022), the role of IL-4^+^ ILC2s can be investigated by creating ILC2-specific *Il4*-deficient mice in the future.

## Materials and methods

### Mice

C57BL/6 mice maintained under SPF or GF conditions were purchased from Sankyo Laboratories Japan, SLC Japan, or CLEA Japan. GF mice were bred and maintained within the gnotobiotic animal facility of Keio University School of Medicine or RIKEN Yokohama Institute. G4 and 4C13R mice were kindly provided by Dr. William E. Paul (National Institute of Allergy and Infectious Diseases, National Institutes of Health; Hu-Li et al., 2001; Roediger et al., 2013). *IL17Rb*^*−/−*^ mice were kindly provided by Dr. Hiroshi Watarai (RIKEN IMS; Watarai et al., 2012). *IL-33*^*−/−*^ mice were purchased from RIKEN BRC (Oboki et al., 2010). *Tslpr*^*−/−*^ mice (BALB/c background) mice were kindly provided by Dr. Steven F. Ziegler (Carpino et al., 2004). *HS2*^−/−^ and *CNS2*^−/−^ mice were generated and characterized in the Masato Kubo lab previously (Harada et al., 2012; Tanaka et al., 2011). *Batf*^−/−^ mice (Schraml et al., 2009) were purchased from the Jackson Laboratories and crossed with Foxp3 reporter (*Foxp3*^hCD2^) mice (Komatsu et al., 2009). *Batf*^−/−^ mice were compared with their *Batf*^+/+^ *Foxp3*^hCD2^ littermates. Formula diets lacking vitamins, minerals, cornstarch, soy oil, or cellulose were purchased from Oriental Yeast. The AIN93G diet was used as a control diet. For supplementation with VB1, thiamine hydrochloride (Nacalai Tesque, Inc) was dissolved in PBS and introduced into mice by intraperitoneal (i.p.) injection at a dose of 200 mg/kg/d or through drinking water at a concentration of 350 μg/ml. For treatment with 6-AN, mice were i.p. injected with 6-AN (10 mg/kg; MP Biomedicals) or an equivalent volume of PBS on days 0 and 4 and euthanized on day 6. For treatment with lactic acid, mice were treated with drinking water supplemented with 0, 5, or 50 mM lactic acid (Nacalai Tesque, Inc.) for 5 wk before sacrifice. To investigate the effect of IL-25 or IL-33 on the development of colonic and lung ILC2s, mice were i.p. injected with recombinant IL-25 (rIL-25) or IL-33 (rIL-33; both from R&D Systems) in PBS daily for 3 d at a dose of 200 ng/mice/d (Huang et al., 2015) and euthanized on day 4. All animal experiments were approved by the Keio University Institutional Animal Care and Use Committee and RIKEN Yokohama Institute.

### Isolation and flow cytometric analysis of intestinal lymphocytes and tuft cells

To analyze intestinal lymphocytes and epithelial cells, intestines were opened longitudinally and washed with PBS to remove luminal contents. All samples were incubated in 20 ml Hanks’ balanced salt solution (HBSS) containing 5 mM EDTA for 20 min at 37°C in a shaking water bath to remove epithelial cells. After vigorous vortexing, colonic epithelial cells released into suspension were centrifuged, immediately frozen in liquid nitrogen, and stored at −80°C until further analysis. An aliquot of epithelial cells was washed with 10 ml of HBSS containing 5 mM EDTA, resuspended in 5 ml of 20% Percoll (GE Healthcare), and underlaid with 2.5 ml of 40% Percoll in a 15 ml Falcon tube to isolate colonic tuft cells. After the epithelial cells were removed, the muscle layer and adipose tissue were removed manually using forceps. The remaining LP layer was cut into small pieces and incubated in 10 ml of RPMI 1640 containing 4% fetal bovine serum, 0.5 mg/ml collagenase D (Roche), 0.5 mg/ml dispase (Gibco), and 40 μg/ml DNase I (Roche) for 45 min at 37°C in a shaking water bath. The digested tissues were washed with 10 ml of HBSS containing 5 mM EDTA, resuspended in 5 ml of 40% Percoll (GE Healthcare), and underlaid with 2.5 ml of 80% Percoll in a 15 ml Falcon tube. Percoll gradient separation was performed using centrifugation at 900 × *g* for 30 min at 25°C. The fraction containing lymphocytes was collected from the interface of the two layers and washed with RPMI 1640 containing 10% FBS. For cytokine detection, the cells were stimulated with 50 ng/ml PMA and 750 ng/ml ionomycin (both from Sigma-Aldrich) in the presence of GolgiStop (BD Biosciences) at 37°C for 3.5 h. After labeling with Ghost Dye 780, the cells were permeabilized and stained with antibodies against GATA3 (AF488; BD Biosciences), Thy1.2 (PE-Cy7; BioLegend), leukocytes lineage markers (CD3ε [BV605; BioLegend], CD4 [BV605; BioLegend], CD11b [BV605; BioLegend], CD11c [BV605; BioLegend], Gr-1 [BV605; BioLegend], CD19 [BV605; BioLegend], TER119 [BV605; BioLegend], and NK1.1 [BV605; BioLegend]), IL-4 (PE or BV421; BioLegend), IL-5 (BV510 or PE; BioLegend), KLRG1 (BV510 or Alexa Fluor 647; BioLegend), ST2 (Alexa Fluor 488 or APC; BioLegend), IL-17RB (PE; BioLegend), IL-13 (PE; BioLegend), and RORγt (PE; BioLegend) using the Foxp3/Transcription Factor Staining Buffer Kit (eBioscience) as per the manufacturer’s instructions. ILC2 cells were defined as the Thy1.2^+^Lineage^−^GATA3^+^ population within the live-cell gate. For epithelial cell staining, the cells were labeled with Ghost Dye 780 (Tonbo Biosciences) and then stained with anti-CD45 (103134; BioLegend), EPCAM (118214; BioLegend) and DCLK1 (primary antibody, rabbit anti-DCLK1, Abcam ab31704; secondary antibody, goat anti-rabbit IgG-AF488, Life Technologies). Tuft cells were defined as the EpCAM^+^CD45^−^DCLK1^+^ population within the live-cell gate. All data were collected on a BD LSRFortessa or FACSAria IIIu (BD Biosciences) instrument and analyzed using the Flowjo software (TreeStar).

### RNA-sequencing

For RNA-sequencing analysis, an RNA library was prepared using a NEBNext Ultra RNA Library Prep Kit for Illumina (New England Biolabs) according to the manufacturer’s instructions. After assessing the library quality, sequencing was conducted on a HiSeq 1500 system (Illumina) using single-ended 50-bp reads. The sequenced reads were mapped to the mouse reference genome (mm9, NCBI build 37) and normalized to fragments per kilobase per million reads (FPKM) values using the Tophat and Cufflinks software pipeline. The heatmaps in Fig. 2 A show the relative abundance (Z-score) of genes whose expressions were upregulated (>twofold, FPKM value ≥ 5) in colonic ILC2s as compared with pulmonary ILC2s isolated from SPF mice. The heatmaps in Fig. 5 C show the comparable relative abundance (Z-score) of genes listed in Fig. 2 A in colonic ILC2s from SPF and GF mice.

### RT-qPCR analysis

Total RNA was isolated from lymphocytes, epithelial cells, and organoids using the TRIzol reagent (Invitrogen) following the manufacturer’s instructions. For qPCR analysis, cDNA was synthesized using ReverTra Ace Master Mix (TOYOBO), and qPCR was performed using the Thunderbird SYBR qPCR Mix (TOYOBO) on a LightCycler 480 (Roche). The following primer pairs were used: *Gapdh*, 5ʹ-CTC​ATG​ACC​ACA​GTC​CAT​GC-3ʹ and 5ʹ-CAC​ATT​GGG​GGT​AGG​AAC​AC-3ʹ; *Actb*, 5ʹ-AGC​CAG​ACC​GTC​TCC​TTG​TA-3ʹ and 5ʹ-TAG​AGA​GGG​CCC​ACC​ACA​C-3ʹ; *Il1a*, 5ʹ-GGT​TAA​ATG​ACC​TGC​AAC​AGG​A-3ʹ and 5ʹ-GGC​TGG​TCT​TCT​CCT​TGA​GC-3ʹ; *Il1b*, 5ʹ-GTG​GAC​CTT​CCA​GGA​TGA​GG-3ʹ and 5ʹ-CGG​AGC​CTG​TAG​TGC​AGT​TG-3ʹ; *Il2*, 5ʹ-CAA​GCT​CTA​CAG​CGG​AAG​CA-3ʹ and 5ʹ-GAG​CAT​CCT​GGG​GAG​TTT​CA-3ʹ; *Il3*, 5ʹ-CCA​GGG​GTC​TTC​ATT​CGA​GA-3ʹ and 5ʹ-CGG​TTC​CAC​GGT​TAG​GAG​AG-3ʹ; *Il4*, 5ʹ-TCA​TCG​GCA​TTT​TGA​ACG​AG-3ʹ and 5ʹ-CCT​TGG​AAG​CCC​TAC​AGA​CG-3ʹ; *Il5*, 5ʹ-TGA​GAC​GAT​GAG​GCT​TCC​TG-3ʹ and 5ʹ-CAG​TAC​CCC​CAC​GGA​CAG​TT-3ʹ; *Il6*, 5ʹ-TTC​TCT​GGG​AAA​TCG​TGG​AAA-3ʹ and 5ʹ-TGC​AAG​TGC​ATC​ATC​GTT​GT-3ʹ; *Il7*, 5ʹ-TTG​CCC​GAA​TAA​TGA​ACC​AA-3ʹ and 5ʹ-GCG​AGC​AGC​ACG​ATT​TAG​AA-3ʹ; *Il9*, 5ʹ-ACA​GCT​GAC​CAA​TGC​CAC​AC-3ʹ and 5ʹ-GGT​CTG​GTT​GCA​TGG​CTT​TT-3ʹ; *Il10*, 5ʹ-AGA​GAA​GCA​TGG​CCC​AGA​AA-3ʹ and 5ʹ-CTC​TTC​ACC​TGC​TCC​ACT​GC-3ʹ; *Il11*, 5ʹ-GGC​TAC​TCC​GCC​GTT​TAC​AG-3ʹ and 5ʹ-CCT​CCT​AGG​ATG​GCA​TGA​GC-3ʹ; *Il12a*, 5ʹ- GAA​GAC​ATC​ACA​CGG​GAC​CA-3ʹ and 5ʹ-CAG​CTC​CCT​CTT​GTT​GTG​GA-3ʹ; *Il12b*, 5ʹ-TGC​TGC​TCC​ACA​AGA​AGG​AA-3ʹ and 5ʹ-CGT​GAA​CCG​TCC​GGA​GTA​AT-3ʹ; *Il13*, 5ʹ-TGC​CAT​CTA​CAG​GAC​CCA​GA-3ʹ and 5ʹ-GGC​GAA​ACA​GTT​GCT​TTG​TG-3ʹ; *Txlna*, 5ʹ-AGC​TAG​TGG​ACG​CCA​AGC​TC-3ʹ and 5ʹ-CTT​CAT​CAG​CTC​GCA​CAT​CC-3ʹ; *Il15*, 5ʹ-TGC​TCT​ACC​TTG​CAA​ACA​GCA-3ʹ and 5ʹ-CCT​CCA​GCT​CCT​CAC​ATT​CC-3ʹ; *Il16*, 5ʹ-TCC​AAT​GAC​CAA​GAA​ATC​TGC-3ʹ and 5ʹ-GTG​CTC​AGT​GAC​CGA​GTT​GG-3ʹ; *Il17a*, 5ʹ-GTT​CCA​CGT​CAC​CCT​GGA​CT-3ʹ and 5ʹ-ATG​TGG​TGG​TCC​AGC​TTT​CC-3ʹ; *Il17b*, 5ʹ-TGA​CTT​GGT​GGG​ATG​GAC​TG-3ʹ and 5ʹ-CCT​CCC​TTG​CCC​TTT​TCT​TT-3ʹ; *Il17c*, 5ʹ-AGG​AGG​TGC​TGG​AAG​CTG​AC-3ʹ and 5ʹ-CTG​TCT​CAC​GGC​CTG​TCT​TG-3ʹ; *Il17d*, 5ʹ-GCG​GCG​CCC​TTA​TTT​ACT​TC-3ʹ and 5ʹ-TGC​AGC​GTG​TGG​TGG​AA-3ʹ; *Il17f*, 5ʹ-CAA​CCA​AAA​CCA​GGG​CAT​TT-3ʹ and 5ʹ-CAG​CGA​TCT​CTG​AGG​GGA​AC-3ʹ; *Il18*, 5ʹ-TGG​CTG​CCA​TGT​CAG​AAG​AC-3ʹ and 5ʹ-CAG​TGA​AGT​CGG​CCA​AAG​TT-3ʹ; *Il19*, 5ʹ-AGG​AAG​CCA​CCA​ATG​CAA​CT-3ʹ and 5ʹ-GTC​AGG​CTG​CAG​GAG​TTT​CC-3ʹ; *Il20*, 5ʹ-AGC​CTC​GCC​AAC​TCC​TTT​CT-3ʹ and 5ʹ-TCT​TCC​CCA​CAA​TGA​CAT​GC-3ʹ; *Il21*, 5ʹ-GCC​AGA​TCG​CCT​CCT​GAT​TA-3ʹ and 5ʹ-CAA​AAG​CTG​CAT​GCT​CAC​AG-3ʹ; *Il22*, 5ʹ-GGT​GAC​GAC​CAG​AAC​ATC​CA-3ʹ and 5ʹ-CCA​ATC​GCC​TTG​ATC​TCT​CC-3ʹ; *Il23a*, 5ʹ-TGG​TTG​TGA​CCC​ACA​AGG​AC-3ʹ and 5ʹ-CAG​GCT​CCC​CTT​TGA​AGA​TG-3ʹ; *Il24*, 5ʹ-ACA​GAT​TCT​CCC​CTG​CCT​GA-3ʹ and 5ʹ- CAG​AAG​GCC​TCC​CAC​AGT​TC-3ʹ; *Il25*, 5ʹ-TCC​AGT​CAG​CCT​CTC​TCA​GA-3ʹ and 5ʹ-CAA​GAA​TGC​AAC​AGC​CTG​GT-3ʹ; *Il27*, 5ʹ-TCT​CGA​TTG​CCA​GGA​GTG​AA-3ʹ and 5ʹ-GAA​GGG​CCG​AAG​TGT​GGT​AG-3ʹ; *Ifnl2*, 5ʹ-TCC​CAG​TGG​AAG​CAA​AGG​AT-3ʹ and 5ʹ-GGA​AGA​GGT​GGG​AAC​TGC​AC-3ʹ; *Ifnl3*, 5ʹ-TCC​CAG​TGG​AAG​CAA​AGG​AT-3ʹ and 5ʹ-GGA​GAT​GAG​GTG​GGA​ACT​GC-3ʹ; *Il31*, 5ʹ-GTG​CCC​CAA​TAT​CGA​AGG​AA-3ʹ and 5ʹ-GCT​GAA​ACA​CGG​CAG​CTG​TA-3ʹ; *Il33*, 5ʹ-AGA​CTC​CGT​TCT​GGC​CTC​AC-3ʹ and 5ʹ-CCC​GTG​GAT​AGG​CAG​AGA​AG-3ʹ; *Il34*, 5ʹ-GGG​CAA​GCT​GCA​GTA​CAA​GA-3ʹ and 5ʹ-CGA​AGC​TCT​CGC​TCA​CTC​AC-3ʹ; *Ebi3*, 5ʹ-AGA​GCC​ACA​GAG​CAT​GTC​CA-3ʹ and 5ʹ-CAC​GGG​ATA​CCG​AGA​AGC​AT-3ʹ; *Il36rn*, 5ʹ-CTG​ACT​GCC​GAA​GCT​TCC​TT-3ʹ and 5ʹ-CCC​ACA​AAG​CAT​CCA​TCA​GA-3ʹ; *Il36a*, 5ʹ-TGT​GTG​GAT​CCT​GCA​GAA​CA-3ʹ and 5ʹ-ATA​TTG​GCA​TGG​GAG​CAA​GG-3ʹ; *Il36b*, 5ʹ-GTT​GAG​ATG​GAG​GGC​AAA​CC-3ʹ and 5ʹ-GGA​GCC​CTC​TAT​GCC​ATG​AT-3ʹ; *Ifna4*, 5ʹ-TCC​ATC​AGC​AGC​TCA​ATG​AC-3ʹ and 5ʹ-TAT​GTC​CTC​ACA​GCC​AGC​AG-3ʹ; *Ifnb1*, 5ʹ-CCC​TAT​GGA​GAT​GAC​GGA​GA-3ʹ and 5ʹ-ACC​CAG​TGC​TGG​AGA​AAT​TG-3ʹ; *Ifng*, 5ʹ-GCG​TCA​TTG​AAT​CAC​ACC​TG-3ʹ and 5ʹ-CTG​GAC​CTG​TGG​GTT​GTT​GA-3ʹ; *Dclk1*, 5ʹ-CAA​GCC​AGC​CAT​GTC​GTT​C-3ʹ and 5ʹ-TTC​CTT​TGA​AGT​AGC​GGT​CAC-3ʹ; *Pou2f3*, 5ʹ-AGA​GAA​TCA​ACT​GCC​CCG​TG-3ʹ and 5ʹ-GGA​AGG​CAC​GAC​TCT​CTT​CC-3ʹ; *Trpm5*, 5ʹ-TAT​GGC​TTG​TGG​CCT​ATG​GT-3ʹ and 5ʹ-ACC​AGC​AGG​AGA​ATG​ACC​AG-3ʹ; *Tslp*, 5ʹ-CGT​GAA​TCT​TGG​CTG​TAA​ACT-3ʹ and 5ʹ-GTC​CGT​GGC​TCT​CTT​ATT​CT-3ʹ; *ThTr1*, 5ʹ-GTT​CCT​CAC​GCC​CTA​CCT​TC-3ʹ and 5ʹ-GCA​TGA​ACC​ACG​TCA​CAA​TC-3ʹ; *ThTr2*, 5ʹ-TCA​TGC​AAA​CAG​CTG​AGT​TCT-3ʹ and 5ʹ-ACT​CCG​ACA​GTA​GCT​GCT​CA-3ʹ.

### Tuft cell staining

For tuft cell staining, intestinal tissues were flushed with PBS and fixed in 4% paraformaldehyde overnight. Tissues were washed with PBS and incubated in 30% (wt/vol) sucrose overnight at 4°C. Colon samples were then coiled into ‘‘Swiss rolls’’ and embedded in Optimal Cutting Temperature Compound (Tissue-Tek) and sectioned at 14 μm on a Microm HM550 cryostat (Thermo Fisher Scientific). The tissues were incubated in 2% goat serum for 1 h, followed by an incubation with the primary antibodies (anti-DCLK1, ab31704; Abcam) and DAPI (Thermo Fisher Scientific) overnight. The tissues were then incubated with goat anti-rabbit IgG F(ab’)2-AF488 secondary antibodies for 1 h and then mounted with ProLong gold antifade reagent (Thermo Fisher Scientific) on slides. Images were acquired with KEYENCE (BZ-X810) using a 10× NA 0.45 lens. Tuft cell proportions were calculated using ImageJ software to manually quantify DCLK1^+^ cells per crypt–villus axis. Four images were analyzed for each replicate.

### Organoid culturing

Intestinal organoids were generated from crypts isolated from the colon of SPF C57BL/6 mice as previously described (Fujii et al., 2018). Briefly, the mouse colon was cut into 1 mm pieces, washed three times in cold PBS, and incubated with 5 mM EDTA for 30 min at 4°C with rocking. Following vigorous pipetting, a 100 μm cell strainer was used to select the fractions enriched with desirable crypts. The isolated crypts were embedded in Matrigel (Corning) and cultured in the following organoid growth medium: advanced DMEM/F-12 (Gibco) or modified advanced DMEM/F-12 without thiamine HCl supplemented with 10 mM HEPES, 2 mM GlutaMAX, 100 U/ml penicillin, 100 μg/ml streptomycin, 20% Afamin/Wnt3a CM (MBL), 50 ng/ml mouse recombinant EGF (Gibco), 100 ng/ml mouse recombinant noggin (Peprotech), 1 μg/ml human recombinant R-spondin 1 (R&D Systems), 500 nM A83-01 (Tocris), 1× N2 supplement (Gibco), 1× B-27 supplement (Gibco), 10 μM Y-27632 (Fujifilm Wako Pure Chemical Corporation), and 1 mM N-acetyl-L-cysteine (Sigma-Aldrich). The organoids were passaged once a week by physical dissociation using fire-polished Pasteur pipettes and TrypLE Express (Thermo Fisher Scientific). To evaluate the effect of VB1 and IL-4 on tuft cell differentiation, the organoids were grown in organoid growth medium in the presence or absence of thiamine HCl, then cultured with or without recombinant IL-4 (400 ng/ml, 214-14; PeproTech) for 2 d. EGF was removed from the growth medium to enhance cell differentiation for the last 2 d. For whole-mount immunostaining of organoids, organoids were fixed in 4% paraformaldehyde O/N at 4°C. After washing three times with 0.03% Triton-X100 in PBS, the organoids were permeabilized and blocked with 0.5% Triton-X100, 5% normal goat serum, and 1% BSA in PBS for 2 h at room temperature with gentle shaking, followed by a 2-d incubation with anti-Pou2f3 (sc-293402; 1:100; Santa Cruz Biotechnology) at 4°C. The organoids were washed three times with 0.03% Triton-X100 in PBS and stained with Alexa Flour 647-labeled anti-mouse antibody (1:500; BioLegend) O/N at 4°C. Nuclear counterstaining was performed using DAPI (5 μg/ml; BioLegend) in PBS for 30 min at RT before imaged under a TCS SP5 (Leica) confocal microscope. Images were analyzed by ImageJ.

### Mucus visualization

For mucus visualization, a transverse sample was taken at the same position within colonic tissue, fixed with methanol-Carnoy’s solution and embedded in paraffin. Colonic sections were incubated with blocking buffer (1% BSA, 2% FBS, 0.05% Tween20 in PBS) at room temperature for 60 min and stained with rabbit anti-MUC2 monoclonal antibody (1:1,000; Santa Cruz Biotechnology) for 120 min, followed by Alexa Fluor 546-labeled goat anti-rabbit IgG (1:500, Life Technologies) in blocking buffer for 60 min. All sections were counterstained with 4,6-diamidino-2-phenylindole (1:5,000; DAPI; Dojindo), mounted with Fluoromount/Plus (Diagnostics BioSystems) and visualized under a TCS SP5 (Leica) confocal microscope. The thickness of the colonic mucus layer and the number of goblet cells were measured using ImageJ software.

### TNBS-induced colitis

C57BL/6 SPF adult mice were fed with a VB1-deficient or control diets for 3 wk. 2,4,6-Trinitrobenzene sulphonic acid (TNBS; Sigma-Aldrich, 2.0 mg in 50% ethanol [EtOH]) was intracolonically administered to anaesthetized mice using a thin round-tip needle. The needle tip was inserted 4 cm proximal to the anal verge, and mice were held in a vertical position for 30 s after the injection. The mice were observed daily and were sacrificed on day 2 after TNBS administration. To evaluate the severity of colitis, colons were fixed with 4% paraformaldehyde, embedded in paraffin, sectioned, and stained with hematoxylin and eosin. Images were acquired with a KEYENCE (BZ-X810) using a 10× NA 0.45 lens. The degree of inflammation in the distal part of the colon was graded from 0 to 4 as follows: 0, normal; 1, ulcer with cell infiltration limited to the mucosa; 2, ulcer with limited cell infiltration in the submucosa; 3, focal ulcer involving all layers of the colon; 4, multiple lesions involving all layers of the colon, or necrotizing ulcer larger than 1 mm in length.

### Statistical analysis

All statistical analyses were performed using GraphPad Prism software (GraphPad Software, Inc.) or JMP software v.12 (SAS Institute, Inc.) with two-tailed unpaired Student’s *t* test (parametric), and one-way ANOVA followed by Tukey’s post-hoc test (three or more groups, parametric).

### Online supplemental material

The supplementary information shows IL-4^+^ ILC2s in the small intestine of *Il17rb*^−/−^ mice (Fig. S1), the expression of VB1 transporters by intestinal epithelial cells (Fig. S2), and influences of VB1-deficiency on IL-13 expression by ILC2s (Fig. S3).

## Data Availability

The raw RNAseq data that support the findings of this study have been deposited in the DNA Data Bank of Japan (DDBJ) SRA under the accession no. DRA016167 (Fig. 2 A and Fig. 5 C) and the accession no. DRA016166 (Fig. 7 B). All FPKM values were deposited in Dryad. Other data are available from the corresponding author upon reasonable request.
